# Facing successfully high mental workload and stressors: An fMRI study

**DOI:** 10.1002/hbm.25703

**Published:** 2021-11-05

**Authors:** Mickaël Causse, Evelyne Lepron, Kevin Mandrick, Vsevolod Peysakhovich, Isabelle Berry, Daniel Callan, Florence Rémy

**Affiliations:** ^1^ ISAE‐SUPAERO, Université de Toulouse Toulouse France; ^2^ Centre de Recherche Cerveau et Cognition Université de Toulouse UPS and CNRS Toulouse France; ^3^ ATR Neural Information Analysis Laboratories Kyoto Japan

**Keywords:** acute stress, auditory stressors, fMRI, heart rate, mental effort, mental workload, pupil diameter

## Abstract

The present fMRI study aimed at highlighting patterns of brain activations and autonomic activity when confronted with high mental workload and the threat of auditory stressors. Twenty participants performed a complex cognitive task in either safe or aversive conditions. Our results showed that increased mental workload induced recruitment of the lateral frontoparietal executive control network (ECN), along with disengagement of medial prefrontal and posterior cingulate regions of the default mode network (DMN). Mental workload also elicited an increase in heart rate and pupil diameter. Task performance did not decrease under the threat of stressors, most likely due to efficient inhibition of auditory regions, as reflected by a large decrement of activity in the superior temporal gyri. The threat of stressors was also accompanied with deactivations of limbic regions of the salience network (SN), possibly reflecting emotional regulation mechanisms through control from dorsal medial prefrontal and parietal regions, as indicated by functional connectivity analyses. Meanwhile, the threat of stressors induced enhanced ECN activity, likely for improved attentional and cognitive processes toward the task, as suggested by increased lateral prefrontal and parietal activations. These fMRI results suggest that measuring the balance between ECN, SN, and DMN recruitment could be used for objective mental state assessment. In this sense, an extra recruitment of task‐related regions and a high ratio of lateral versus medial prefrontal activity may represent a relevant marker of increased but efficient mental effort, while the opposite may indicate a disengagement from the task due to mental overload and/or stressors.

## Graphical abstract

We highlighted patterns of brain activations and autonomic activity when confronted with high mental workload and auditory stressors. Mental workload induced recruitment of the fronto‐parietal executive control network (ECN), along with disengagement of the default mode network (DMN). Task performance did not decrease under stressors, most likely due to efficient emotional regulation mechanisms through control from dorsal brain areas.

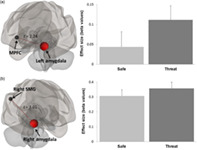

## 1 INTRODUCTION

A fine‐grained understanding of how the brain copes with an important mental workload or a stressful situation is a major issue to promote human performance in a challenging environment. Complex and safety‐critical activities, such as piloting an airplane or operating a nuclear power plant, can lead to a drastic and simultaneous increase of both mental workload and acute stress. These effects originate from a combination of task complexity (Borghini, Astolfi, Vecchiato, Mattia, & Babiloni, 2014; Causse, Chua, Peysakhovich, Del Campo, & Matton, 2017; Durantin, Gagnon, Tremblay, & Dehais, 2014) and the potentially fatal consequence of errors (Kilic & Ucler, 2019). In this context, the maintenance of optimal cognitive performance is a constant challenge. According to several authors, high mental workload (Causse, Peysakhovich, & Fabre, 2016) and acute stress (Arnsten, 2009; Qin, Hermans, Marle, Luo, & Fernández, 2009; Schoofs, Wolf, & Smeets, 2009; Starcke, Wiesen, Trotzke, & Brand, 2016) may both result in transient cognitive deficits, that is, impairments in executive functions and working memory (WM) in particular. Meanwhile, cerebral compensatory processes may be engaged and contribute to preserving cognitive performance (Fairclough & Mulder, 2012). A primary objective for improving safety in these critical situations is therefore a better understanding of neurophysiological mechanisms involved in either maintenance or deterioration of cognitive performance under challenging conditions.

Enhanced task complexity can result in an increase of mental effort, corresponding to the amount of brain resources/cognitive capacity an individual puts into a task (Galy, Cariou, & Mélan, 2012), which might be indexed via brain activity or autonomic parameters (e.g., level of brain activity and pupil size). Task complexity also likely leads to greater mental workload, often inferred from overt behavior or performance. Mental workload roughly corresponds to the interplay between the demands of the environment (input load), human individual characteristics (capacities) and task performance (output) (Causse et al., 2017). A decrease in cognitive task performance will occur whenever there is a mismatch between environmental demands and individual capabilities, that is, if the workload is too high or too low (Kantowitz & Casper, 1988). Thus, taking into account solely the task characteristics does now allow inferring the level of mental workload in an individual.

In the scientific literature, mental workload and stress are often designated indistinctively with terms like “mental stress” (Hjortskov et al., 2004), probably because their causes and effects can be similar. Also, their occurrence can be concomitant: a task generating an important mental workload can lead to an increase in mental stress because the individual will be overwhelmed by the difficulty (Warm, Parasuraman, & Matthews, 2008) or will feel the situation as emotionally challenging. However, mental workload and stress may reasonably be considered as distinct phenomena (Hidalgo‐Muñoz et al., 2018). According to Gaillard (1993), it is possible to work quite hard on difficult and complex tasks, even under unfavorable conditions, without cognitive strain or adverse physiological effects. High task demands can be met by mobilizing extra cognitive resources. In contrast, mental stress is regarded by Gaillard as a state, in which cognitive resources allocation is inefficient and disturbed by negative emotions. In other words, a high level of mental workload does not necessarily elicit a high stress level, and a high stress level may also occur when mental workload is low. Altogether, these results underline the difficulty to disentangle cognitively‐demanding from acute stress situations, as well as the importance of determining physiological mechanisms supporting both these situations and their possible co‐occurrence.

Complex physiological and brain mechanisms take place with both high mental workload and acute stress. The executive control network (ECN) is of particular importance regarding cognitive load management. The ECN comprises the lateral and medial parts of the dorsal prefrontal cortex (PFC), premotor regions and the lateral posterior parietal cortex. Its role is crucial for sustained and selective attention, cognitive flexibility, WM, and decision making in goal‐directed behaviors (Corbetta & Shulman, 2002). During effortful tasks, increased demands on executive functions and WM enhance activity in the fronto‐parietal network of the ECN and in the dorsal anterior cingulate cortex (ACC) (Ayaz et al., 2012; Dosenbach, Fair, Cohen, Schlaggar, & Petersen, 2008; Engström, Karlsson, Landtblom, & Craig, 2015; Khachouf, Chen, Duzzi, Porro, & Pagnoni, 2017; Mulert et al., 2008; Owen, McMillan, Laird, & Bullmore, 2005; Power & Petersen, 2013; Schmidt et al., 2009; Shenhav, Botvinick, & Cohen, 2013; Shenhav et al., 2017). In particular, dorsolateral PFC (DLPFC) activity increases linearly with WM load (Braver et al., 1997), suggesting that the DLPFC region of the ECN represents a reliable proxy measure of mental workload (Mandrick, Peysakhovich, Rémy, Lepron, & Causse, 2016; Parent, Peysakhovich, Mandrick, Tremblay, & Causse, 2019). A study of Shen et al. (2020) showed that connection strength between regions of the ECN is strongly correlated with executive function performance. High mental workload does not only elicit enhanced activity in the ECN: several brain regions can show reduced activity during the performance of demanding tasks, including the medial prefrontal cortex, the posterior cingulate cortex, and the precuneus. This set of regions belong to the default mode network (DMN) (Fransson & Marrelec, 2008; Greicius, Krasnow, Reiss, & Menon, 2003; Raichle et al., 2001; Ward et al., 2014).

ECN activity is influenced not only by mental workload but also by acute stress (Arnsten, Wang, & Paspalas, 2012; Hermans, Henckens, Joëls, & Fernández, 2014; Van Oort et al., 2017). Depending on its level, stress may either improve or deteriorate the ECN function. Moderate levels of stress may induce a more efficient PFC function (Arnsten, 2009; Yuen et al., 2009), while a highly stressful condition would induce an impaired PFC function (Arnsten et al., 2012). Other large brain networks seem to be sensitive to increased stress levels. During episodes of high levels of stress, engagement of the amygdala and related limbic structures has been reported, with significant activity in the anterior insula, the dorsal ACC, the hippocampus, and the hypothalamus (Hermans et al., 2014; Van Oort et al., 2017). This set of regions has been described as the salience network (SN). Involvement of the SN may sustain the orientation of attention toward salient information to promote threat detection. Accordingly, SN activity decreases under low or moderate levels of stress (Pruessner et al., 2008), while predominant involvement of the SN is observed under highly stressful situations.

Few imaging studies have investigated the combined effects of cognitive workload and stressors. Under stress, activity in lateral PFC regions of the ECN implied in the cognitive task either increases (Clarke & Johnstone, 2013; Porcelli et al., 2008), remains unchanged (Cousijn, Rijpkema, Qin, van Wingen, & Fernández, 2012) or decreases (Qin et al., 2009). Accordingly, the PFC could have a critical role in mediating stress influence on cognition (Bogdanov & Schwabe, 2016; Clarke & Johnstone, 2013; Porcelli et al., 2008; Shields, Sazma, & Yonelinas, 2016). Moreover, when acute stress and high cognitive load are combined, a dynamic interplay between ECN, DMN, and SN, possibly driven by the dorsal ACC and the ventral lateral PFC (VLPFC) (Clarke & Johnstone, 2013; Pruessner et al., 2008; Simpson, Snyder, Gusnard, & Raichle, 2001), may enable the reallocation of neural resources. A shift toward the SN and DMN would reduce cognitive efficiency (Qin et al., 2009) and enhance environment scanning at the cost of cognitive performance ensured by the ECN. Conversely, a shift toward the ECN, along with top‐down inhibition of the SN and DMN, may enable preservation of cognitive performance under stress. In previous studies, mental workload was elicited with WM tests such as two‐back letter or digit tasks. These tasks were combined with acute stress induced by an aversive movie (Cousijn et al., 2012; Qin et al., 2009), the threat of shock (Clarke & Johnstone, 2013), or the cold pressor test (Duncko, Johnson, Merikangas, & Grillon, 2009; Porcelli et al., 2008). In these studies, high‐load WM task accuracy was preserved under stress. Preservation of WM performance may pertain to the relatively moderate perceived workload and the involvement of emotion regulation mechanisms. Conversely, other studies have reported reduced performance following exposure to stressors, either psychosocial (Jiang & Rau, 2017; Schoofs, Preuß, & Wolf, 2008) or physical (cold pressor test) (Schoofs et al., 2009). Hence, there is no clear pattern for the influence of stress on cognition, this influence being likely dependent on the height of the cognitive load and the level and nature of stress. In particular, it still remains unclear whether performance on a more demanding cognitive task, relying on the ECN and efficient PFC function, could be maintained under stressors (Clarke & Johnstone, 2013; Porcelli et al., 2008; Schoofs et al., 2008).

In the present fMRI study, we investigated performance and brain activity during a highly difficult task performed under varying levels of task difficulty and the threat of stressors. We introduced a paradigm that reproduces the mentally challenging conditions that operators face during degraded contexts. To achieve this aim, we used the novel Toulouse n‐bask task (TNT) that combines a classical n‐back task with mental arithmetic. The n‐back WM paradigms were consistently shown to imply bilateral DLPFC and dorsal lateral parietal regions. Mental calculation involves a large‐scale network, including lateral and medial prefrontal and motor regions, as well as inferior and superior parietal cortex (Gruber, Indefrey, Steinmetz, & Kleinschmidt, 2001; Klein, Moeller, Glauche, Weiller, & Willmes, 2013; Kong et al., 2005). By combining n‐back WM and mental arithmetic processes, the TNT task was thus thought to rely heavily on an extended fronto‐parietal network encompassing the ECN, and was conceived to mimic the multidimensional high mental workload existing in many safety‐critical occupations such as aircraft piloting (Mandrick et al., 2016). In addition, this novel n‐back task was completely embedded into a threatening or safe context. The threatening context was induced using frequent but unexpected occurrences of aversive auditory stimuli played in parallel with the task. This induction method is potentially more efficient than the use of a punctual emotional induction delivered before the task, whose effects can fade out progressively, for example, when using an emotional movie clip before task execution (Cousijn et al., 2012; Du et al., 2020; Jiang & Rau, 2017; Qin et al., 2009). Also, previous research using physical stressors during task performance may have been confounded by distraction effects due to the salience of the stimulus (Duncko et al., 2009; Porcelli et al., 2008; Schoofs et al., 2009). In this study, we were interested in the experienced stress per se, we thus excluded the time period during which the aversive stimuli were delivered, focusing on the effects of the threat of unpredictable loud unpleasant sounds. Another important aspect of the experimental paradigm was the manipulation of both task difficulty and the presence of stressors while measuring pupil diameter and cardiac activity, both being particularly sensitive to mental effort (Eysenck & Calvo, 1992; Fairclough & Houston, 2004; Gray & Braver, 2002; Peysakhovich, Causse, Scannella, & Dehais, 2015) and stress (DeBeck, Petersen, Jones, & Stickland, 2010; Yao et al., 2016). Such physiological recordings are feasible in situ, for example, in cockpits during flight. Thus concurrent neuroimaging and physiological measures during variable mental load and stress conditions are particularly relevant (Alnæs et al., 2014; Brown et al., 1999; Khachouf et al., 2017; Mandrick et al., 2016; Parent et al., 2019; Wang et al., 2005). We thus investigated how brain activity co‐varied with these two physiological measurements thanks to parametric modulation analyses.

We hypothesized that cognitive performance would deteriorate under high workload and we expected greater involvement of the ECN regions, particularly the DLPFC and the lateral parietal cortex, and deactivations of the DMN, altogether suggesting increased mental workload due to the challenging task. Both heart rate and pupil diameter should be increased. We also expected that performance should be relatively preserved under stress induction thanks to emotional regulation. However we expected that the combination of high task difficulty and stress induction may lead to an overload of the ECN. A marked decrease of task performance and an opposite brain pattern of activations to those observed under the high workload should be observed, with a decrease in the ECN regions along with SN activations. Heart rate and pupil diameter might be either increased or decreased since a disengaging of the task may occur, generating a decline of the sympathetic activity, while the stress could, on the contrary, increase sympathetic activity.

## 2 METHODS AND MATERIALS

### 2.1 Participants

Twenty young, healthy participants (6 females, 14 males; mean age = 24 years, *SD* = 3.9 years and range = 20–34) were involved in this study. All had studied for at least 2 years at university (after completion of their secondary education). Four participants were left‐handed, while the others were right‐handed. The hand dominance did not affect behavioral performance reported in this study. None reported either affective or anxiety disorders or any neurological or cardiovascular disease. None were under any form of medication that might affect the brain or autonomic functions. All participants reported normal auditory acuity and normal or corrected‐to‐normal vision. The study complied with the Declaration of Helsinki for human experimentation and was approved by a National Ethics Board (CPP du Sud‐Ouest et Outre‐Mer IV, no. CPP15‐010b/2015‐A00458‐41). All participants signed a consent form and were paid for their participation. They were informed that they would be submitted to unpleasant loud sounds in the MRI scanner.

### 2.2 Stress induction

Stress (sustained anxiety) induction was performed with the threat of unpredictable loud unpleasant sounds, such as blackboard scratching, plate scratching with a fork, dentist's drill, and so on (Patel et al., 2016). Thirty‐four sounds were selected based on previous works (Grillon et al., 2008; Hirano et al., 2006; Kumar, Forster, Bailey, & Griffiths, 2008; Zald, Hagen, & Pardo, 2002) and on a survey that we conducted on a separate group of 87 participants. According to our survey, the sounds were globally perceived as mildly stressful, uncomfortable, and unpleasant.

During the training session of the present experiment, participants were first asked to estimate the maximum acceptable sound level they could bear (ranging from 80 to 95 dB). This maximum acceptable sound level was later set in the fMRI. They were then exposed to the 34 different sounds. Each participant evaluated these sounds (7‐s duration each) by rating them on a scale from 0 (not aversive) to 10 (highly aversive). Based on the individual ratings, the 17 most unpleasant sounds were selected for each participant to be further presented during the MRI session. The 17 sounds played during the experiment were rated at 7.42 on average across all the participants. Following this, participants were trained to associate “safe” (no aversive sounds) and “threat” (aversive sounds) conditions with colored screens, that is, blue screen and red screen, respectively. During the safe condition, participants had nothing to do except quietly watching the blue colored screen. During the threat condition, participants had to pay attention to several unpleasant loud sounds while watching the red screen.

In the MRI scanner, participants were informed that they would be exposed to unpredictable aversive loud sounds, among those they rated as the most unpleasant, during either active or rest blocks. The onset of sounds was unknown to participants in order to maintain a continuous threat (Grillon et al., 2008; Zald & Pardo, 2002). The aversive sounds occurred randomly during all cognitive tasks and rest conditions and were noncontingent upon the performance of the participant to the task. Each of the 17 aversive sounds was presented once to the participant to prevent habituation. They could be played one time or two consecutive times (without pause between the two occurrences), and never occurred again later in the experiment. The sounds were played via MR‐compatible monitor headphones in stereo mode.

### 2.3 n‐back task

**FIGURE 1:**
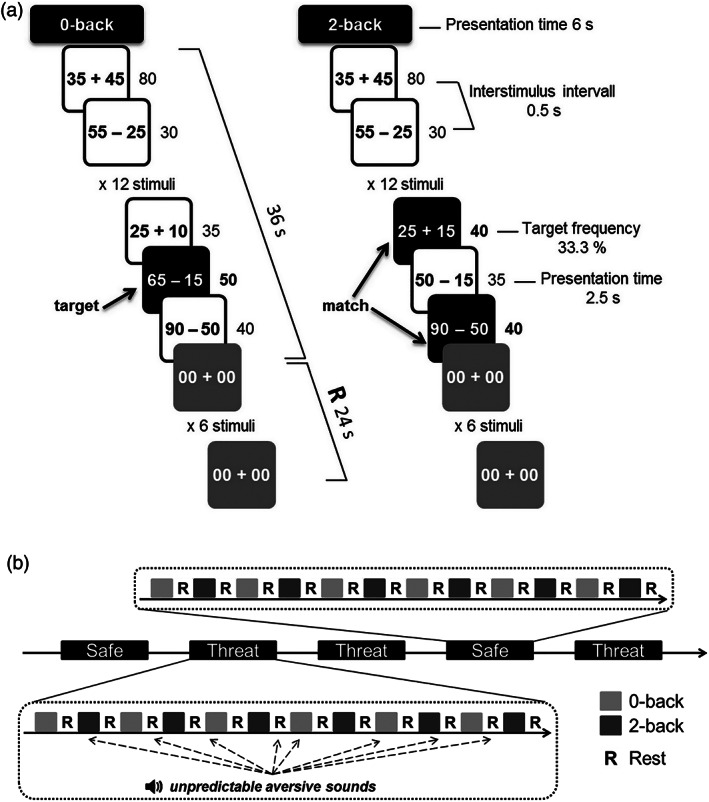
Experimental design. (a) Toulouse n‐back task (TNT). The active blocks consisted of 12 trials and lasted 36 s. They were interleaved with 24‐s rest blocks (R). Participants responded to targets and nontargets by pressing one of two different buttons. The side of the buttons was counterbalanced across participants. (b) experimental timeline. The experiment included five functional runs (two safe runs without any sounds and three threat runs with the possible occurrence of aversive sounds), presented in a counterbalanced order. TNT difficulty levels (0‐back and 2‐back) were counterbalanced and alternated with rest periods. Unpredictable aversive loud sounds were presented randomly during threat runs and could occur during rest and active blocks

The Toulouse n‐back Task (TNT) was implemented in MATLAB (MathWorks) using the Psychophysics Toolbox extensions (Brainard, 1997; Kleiner, Brainard, & Pelli, 2007; Pelli & Vision, 1997). The task is described in detail in a previous publication (Mandrick et al., 2016). The task was developed to combine a classical n‐back task with mental arithmetic. Instead of memorizing and comparing unique items, as in the classical n‐back task, the participants had to memorize and to compare the results of arithmetic operations, computed beforehand. Arithmetic operations were either additions or subtractions. All numbers were multiples of five (e.g., 15 + 40, 90–35). The arithmetic operations (trials) were presented for 2.5 s, followed by an interstimulus‐interval of 0.5 s. Volunteers were required to compute the result of the arithmetic operations and compare it with either a fixed number (0‐back) or the result obtained two trials before (2‐back). In the 0‐back condition, the “target” fixed number was “50.” Participants were therefore asked to press a specific button when the result of the operation was 50. In the 2‐back condition, the participants were asked to press the button whenever the result of the arithmetic operation was identical to the one presented two trials ago (“match”), see Figure 1a.

### 2.4 Procedure

The entire protocol lasted approximately 3 hr. Before scanning, participants performed the training session, during which they were exposed to the auditory stressors further used in the MRI. They were also trained on the TNT for the two levels of task difficulty, that is, 0‐back and 2‐back. This cognitive training included at least one block for the 0‐back condition and three blocks for the 2‐back condition, until participants felt comfortable with the TNT. Furthermore, the training was successively performed in both the safe and the threat conditions. In the MRI, the TNT was presented using a video projector on a translucent screen placed above the head of the participant. He or she viewed the stimuli through a mirror mounted on the head coil above their eyes. Head movement was restricted using foam cushions. All participants underwent five functional runs (apart from two participants, who performed only four runs due to technical problems). The five functional runs consisted of two safe runs and three threat runs with the possible occurrence of aversive sounds, see Figure 1b. We chose to design a within‐subject study (all the participants underwent safe and aversive conditions with concomitant n‐back task). Indeed, we expected rather large intersubject variability in cognitive performance on the complex n‐back calculation task (Mandrick et al., 2016). Therefore, comparing TNT performance in two groups of participants with a between‐subject design may have confounded our results regarding stress effects on cognition. Moreover, similar studies have been previously conducted with no visible effect of stress persistence across stressful and safe alternate sessions (Clarke & Johnstone, 2013; Cousijn et al., 2012). The order of the safe and threat runs was counterbalanced across participants. Half of the participants started each run with the 0‐back level and the other half with the 2‐back level. The TNT was presented with a blocked design. Each functional run included 12 cycles of alternating 0‐back and 2‐back 36‐s active blocks interleaved by 24‐s rest blocks. Within each active block, a series of 12 arithmetic operations (trials) were presented to the participant. The participant was given a 2‐button response box (one button for “target,” the other for “no target”) and was asked to respond as quickly as possible. Each active block contained four targets in random positions. Active blocks were preceded by an instruction cue lasting 6 s. The instruction cue informed the participant about the next n‐back level (0‐back or 2‐back) and the screen color background indicated whether it will be a safe (blue screen) or a threat (red screen) condition. The color was not visible during the active blocks, arithmetic operations were displayed in the center of a gray background. During the rest blocks, “00 + 00” operations were presented and the participant did not give any response. The MRI scanning session lasted about 1 hr and 15 min.

### 2.5 MRI data acquisition

MRI data were acquired on a Philips Achieva 3‐T scanner (Philips, Best, The Netherlands) at the Toulouse Neuroimaging Center technical platform (referred to as ToNIC Inserm UMR 1214), using a 32‐element SENSE head coil. Each of the five functional runs included successive acquisitions of 285 whole‐brain T2*‐weighted echo planar images with blood oxygenation level‐dependent contrast (EPI‐BOLD) sequence [40 axial slices with ascending acquisition order, repetition time (TR) = 2.60 s, echo time (TE) = 30 ms, 90° flip angle, matrix size = 96 × 96, slice‐thickness = 3 mm with no slice gap, field of view (FOV) 240 × 240 mm^2^]. In addition to the five functional runs, each participant also underwent a high‐resolution 3D anatomical scan for functional overlay and stereotaxic transformation. This scan was acquired using a T1‐weighted 3D magnetization‐prepared rapid gradient‐echo (MP‐RAGE) sequence (TR = 8.1 ms, TE = 3.7 ms, 8° flip‐angle, 170 contiguous sagittal slices with matrix size 240 × 240, FOV 240 × 240 × 170 mm^3^).

### 2.6 Autonomic nervous system measures in the MRI

For heart rate measures, the ECG signal was recorded continuously at 500 Hz throughout MRI scanning with an MR‐compatible pulse oximeter (Philips, Best, The Netherlands) attached to the left index finger. Moreover, the pupil diameter of the left eye was recorded continuously with an MR‐compatible eye‐tracking device (long‐range optic ASL EyeTracker 6000, Applied Science Laboratories, Bedford, Massachusetts) at a sampling rate of 60 Hz. The eye‐tracker was positioned behind the scanner and the translucent screen. A hole with a radius of 3 cm was made at the bottom of the screen so that the camera could monitor the subject's eye on the mirror and the infrared light emitter could illuminate the pupil to assess its size.

### 2.7 Subjective ratings of task difficulty and anxiety

A debriefing session was conducted after MRI scanning. Participants were asked to rate the difficulty of the TNT task and the level of anxiety induced by task difficulty and by the threat of the aversive sounds. The rating was done on a 0–10 scale.

### 2.8 Data analysis

#### 2.8.1 Behavioral data

The mean percentage of correctly reported match/no‐match response (corresponding to performance success) and the mean reaction times were calculated for each participant and in each of the four experimental conditions. In addition, d‐prime was calculated as *z*(hit rate) − *z*(false alarm rate). The three variables were analyzed across participants using repeated‐measures 2 × 2 ANOVA with factors of cognitive load (0‐back, 2‐back) and threatening context (safe, threat).

#### 2.8.2 Autonomic nervous system measure analysis

##### Heart rate

ECG signal was first visually controlled for outliers and artifacts. Signal was processed with the “findpeaks” function of MATLAB 2019. The series of R–R interval times were then derived from the ECG and the mean heart rate was calculated for each active 36 s block. Hear rate was then averaged for each of the four experimental conditions. These mean values were further used as parametric modulators in fMRI first‐level models (see below).

##### Pupil diameter

Pupil diameter signal was processed using home‐made MATLAB scripts. Periods of signal loss and blinking as well as six samples before and after each signal loss period (100 ms at 60 Hz) were linearly interpolated. Trials where the number of interpolated samples exceeded 50%, were excluded from analyses. The signal was low‐passed using a 9‐point moving average filter. The pupil diameter was then averaged over all trials of each active 36‐s block, and then averaged for each of the four experimental conditions. Therefore, the mean pupil diameter values were modulated by both tonic and phasic pupil changes induced by cognitive load and/or threatening context. These mean values were further used as parametric modulators in fMRI first‐level models (see below).

#### 2.8.3 MRI data preprocessing

Image preprocessing and statistical analysis were performed using Statistical Parametric Mapping software (SPM8) (http://www.fil.ion.ucl.ac.uk/spm). Functional images were realigned to the first volume with a six‐parameter rigid‐body transformation and the mean functional image was co‐registered to participant's T1‐weighted MR image. Functional images were corrected for slice acquisition timing. Anatomical images were then segmented based on tissue probability maps of gray matter, white matter and CSF in the standard MNI space. The deformation field used for the segmentation was applied to the T1‐weighted and functional images for normalization into MNI stereotactic space. Functional images were resampled into 2‐mm isotropic voxels. Finally, functional images were spatially smoothed by convolving with an isotropic 3D‐Gaussian kernel (8‐mm full width at half maximum).

#### 2.8.4 MRI data statistical analyses

##### First‐level analyses

For each participant, six experimental conditions were implemented as box‐car functions with an epoch length of 36 s, convolved with the canonical hemodynamic response function. Specifically, individual models included four regressors of interest (corresponding to 0‐back safe, 2‐back safe, 0‐back threat, and 2‐back threat epochs) and two regressors of no interest corresponding to the 0‐back and 2‐back task epochs, during which aversive sounds were presented. These two latter conditions of no interest (12 blocks in total) were modeled to exclude any auditory perception effect or any potential distraction effect of sound, and to selectively tackle the effect of stress related to the expectancy of the unpredictable sounds. Rest epochs were implicitly modeled. In this first model, simple contrasts for each of the four conditions of interest were created, comparing active conditions with resting baseline. Moreover, in order to modulate condition effects by autonomic measures, two additional first‐level models were implemented for each participant, which consisted of the same six box‐car regressors with parametric modulation by either heart rate or pupil diameter. To this aim, heart rate and pupil diameter values were averaged over each 36‐s block (see below) and these mean values were entered as parametric modulators of each condition regressor in two separate models. Brain activity specific to each condition and co‐varying with either heart rate or pupil diameter could therefore be assessed. For each of the latter two models, four simple contrasts were created for modulated active conditions versus resting baseline. All first‐level models furthermore included high‐pass filtering using a cutoff of 1/128 Hz, global intensity normalization and serial correlations correction using a first‐order autoregressive model.

##### Second‐level analyses

For group analyses, individual contrasts were entered into a random‐effects model, using the flexible factorial tool in SPM8. A repeated‐measures 2 × 2 ANOVA model was implemented, with task difficulty (two levels: 0‐back and 2‐back) and threat (two levels: safe and threat) as within‐subject factors. Main effects investigated brain activations (i.e., 2‐back > 0‐back and threat > safe) and brain deactivations (i.e., 2‐back < 0‐back and threat < safe) related with TNT difficulty and level of threat. Moreover, difficulty × threat interaction effects investigated differences in brain mechanisms elicited by cognitive effort, during threat and safe conditions. We implemented 3 s‐level ANOVA models, using individual contrasts derived from first‐level analyses (a) without any parametric modulation, (b) with heart rate parametric modulation, and (c) with pupil diameter parametric modulation. In the group analysis that does not take autonomic modulation into account, activations were investigated at an initial voxel‐level threshold of *p* < .001 uncorrected, with an extent threshold of 10 voxels, and a cluster‐level threshold of *p* < .005 corrected for multiple comparisons (family wise error‐FWE) at the whole‐brain level was applied. We reported only the clusters surviving this correction for all contrasts investigated. For the complementary exploratory group analyses which included autonomic parametric modulation analyses, sensitivity was favored over specificity (Wilke, 2012). Therefore, significance was assumed at *p* < .005, uncorrected for multiple comparisons. Since we were particularly interested in ECN regions, we used SPM's small volume correction (SVC) at specific MNI coordinates in the DLPFC previously established as functionally connected to a pupil‐related network (DiNuzzo et al., 2019).

##### Functional connectivity analysis

Task‐based connectivity analysis was performed using the SPM CONN toolbox (https://www.nitrc.org/projects/conn) to investigate changes in connectivity due to the threat of auditory stressors. For each participant, realignment parameters were entered as first‐level covariates, and preprocessed functional images were denoised (using standard CONN procedures) to remove unwanted motion artifacts prior to calculation of connectivity measures. A band‐pass filter of 0.008–0.09 Hz was applied. ROI‐to‐ROI connectivity estimates were computed from correlations of BOLD signal between seed regions evidencing significant effects during threat with all ROIs from the CONN atlas. At the second‐level, differences in connectivity between threat and safe conditions were analyzed across all participants. Significant changes in connectivity due to threat were assessed at a threshold of *p* < .05 with false discovery rate (FDR) correction.

## 3 RESULTS

### 3.1 Anxiety and task difficulty subjective ratings

**FIGURE 2:**
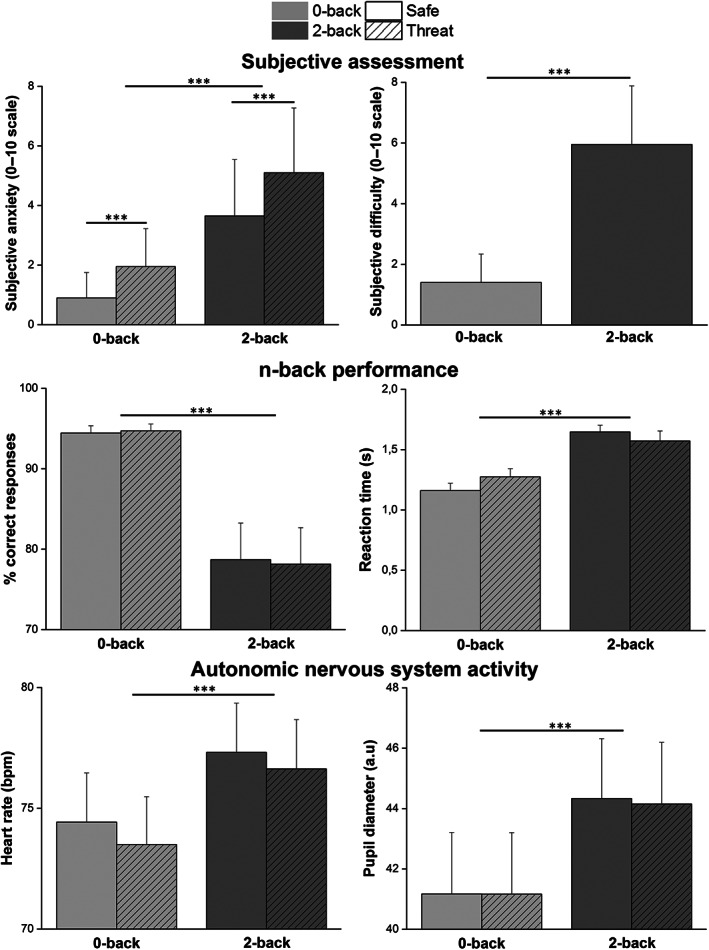
Subjective ratings, behavioral performance and autonomic nervous system activity measures for each level of TNT difficulty (0‐back and 2‐back) and threat conditions (safe and threat), *n* = 20. Top: anxiety (left panel) and task difficulty ratings (both safe and threat conditions averaged, right panel). Middle: percentage of correct responses (left panel) and mean reaction times (right panel). Bottom: Heart rate (left panel) and pupil size (right panel). Error bars are *SEM*. Light gray = 0‐back, medium gray = 2‐back, plain bar = safe, striped bar = threat

The average levels of anxiety induced by the four experimental conditions were 0.90 (*SD* = 0.85) for “0‐back safe,” 1.95 (*SD* = 1.28) for “0‐back threat,” 3.65 (*SD* = 1.90) for “2‐back safe,” and 5.10 (*SD* = 2.17) for “2‐back threat” (Figure 2, top left panel). Subjective anxiety was significantly higher in the 2‐back versus 0‐back condition [*F*(1,19) = 51.71, *p* < .001, *η*_p_^2^ = .73] and in the threat versus safe condition [*F*(1,19) = 15.72, *p* < .001, *η*_p_^2^ = .45]. The interaction term was also significant [*F*(1,19) = 4.75, *p* = .042, *η*_p_^2^ = .20], with increased anxiety when threat and 2‐back conditions were combined.

The subjective levels of difficulty induced by the 0‐back and 2‐back conditions were, respectively, 1.40 (*SD* = 0.94) and 5.95 (*SD* = 1.93) (Figure 2, top right panel). Repeated‐measures ANOVA showed that subjective difficulty was significantly higher in the 2‐back versus 0‐back condition [*F*(1,19) = 133.45, *p* < .001, *η*_p_^2^ = .88].

### 3.2 n‐back performance

#### 3.2.1 Percentage of correct responses

The percentage of correct responses induced by the four experimental conditions were 94.44% (*SD* = 4.05) for “0‐back safe,” 94.71% (*SD* = 3.80) for “0‐back threat,” 78.70% (*SD* = 20.31) for “2‐back safe,” and 78.14% (*SD* = 20.25) for “2‐back threat” (Figure 2, middle left panel). Participants showed lower percentage of correct response with increased difficulty [*F*(1,19) = 18.33, *p <* .001, *η*_p_^2^ = .49]. The threat of unpredictable auditory stressors did not impact accuracy (*p* = .853). The interaction term was not significant (*p* = .502).

#### 3.2.2 d‐prime

The average d‐prime induced by the four experimental conditions was 2.43 (*SD* = 0.22) for “0‐back safe,” 2.48 (*SD* = 0.24) for “0‐back threat,” 1.63 (*SD* = 0.64) for “2‐back safe,” and 1.60 (*SD* = 0.55) for “2‐back threat.” Participants showed lower d‐prime with increased difficulty [*F*(1,19) = 118.31, *p <* .001, *η*_p_^2^ = .86]. The threat of unpredictable auditory stressors did not significantly impact d‐prime values (*p* = .696). The interaction term was not significant (*p* = .270).

#### 3.2.3 Reaction times

The reaction times induced by the four experimental conditions were 1.16 s (*SD* = 0.27) for “0‐back safe,” 1.27 s (*SD* = 0.29) for “0‐back threat,” 1.64 s (*SD* = 0.25) for “2‐back safe,” and 1.57 s (*SD* = 0.35) for “2‐back threat” (Figure 2, middle right panel). Participants showed longer reaction times with increased difficulty [*F*(1,19) = 54.73, *p* < .001, *η*_p_^2^ = .74]. The main effect of threat was not significant (*p* = .528). The difficulty × threat interaction was significant [*F*(1,19) = 7.68, *p* = .012, *η*_p_^2^ = .12], showing an increase of reaction times due to threat in the 0‐back condition and a decrease of reaction times in the 2‐back condition. However, HSD post hoc tests revealed no significant pairwise comparisons between threat and safe conditions.

### 3.3 Autonomic nervous system results

#### 3.3.1 Heart rate

The mean heart rate induced by the four experimental conditions was 74.42 bpm (*SD* = 8.60) for “0‐back safe,” 73.49 bpm (*SD* = 8.38) for “0‐back threat,” 77.31 bpm (*SD* = 8.61) for “2‐back safe,” and 76.63 bpm (*SD* = 8.63) for “2‐back threat” (Figure 2, bottom left panel). Participants showed a higher heart rate with increased task difficulty [*F*(1,19] = 53.69, *p* < .001, *η*_p_^2^ = .74]. The main effect of threat and the interaction term were not significant (*p* = .180 and *p* = .419, respectively).

#### 3.3.2 Pupil diameter

The mean pupil diameter induced by the four experimental conditions was 41.17 mm (*SD* = 7.86) for “0‐back safe,” 41.16 mm (*SD* = 7.58) for “0‐back threat,” 44.33 mm (*SD* = 8.09) for “2‐back safe,” and 44.15 mm (*SD* = 8.11) for “2‐back threat” (Figure 2, bottom right panel). Participants showed higher pupil diameter with increased task difficulty [*F*(1,19) = 60.08, *p* < .001, *η*_p_^2^ = .76]. The main effect of threat and the interaction term were not significant (*p* = .823 and *p* = .490, respectively).

### 3.4 Functional MRI results

#### 3.4.1 Main effect of mental workload

**FIGURE 3:**
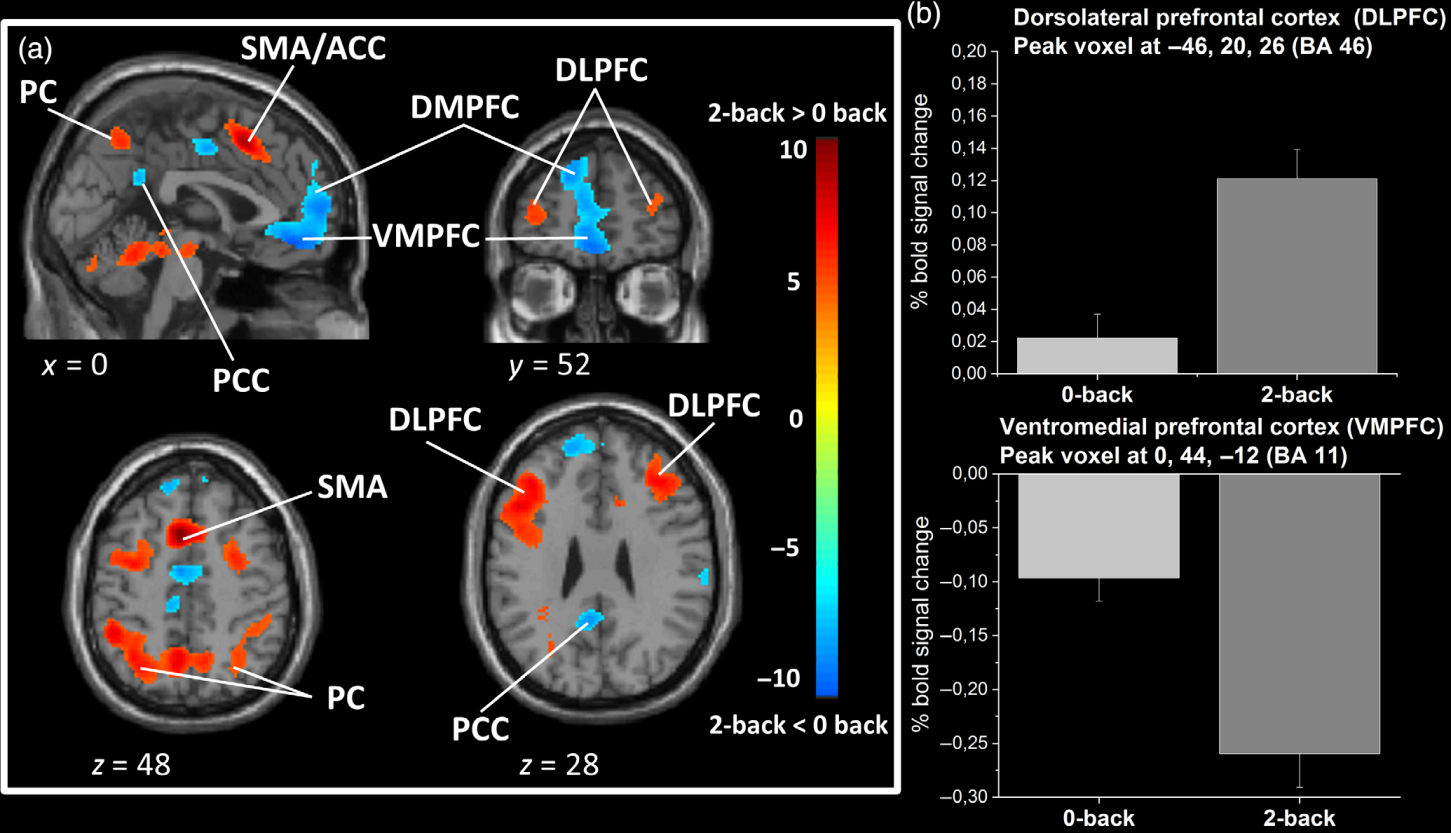
Brain activations and deactivations related to mental workload. (a) Statistical parametric maps illustrating the main effect of mental workload in the TNT. The colored bar indicates the *t*‐value (+10 to −10) of the activation height. Cortical areas evidencing increased (red color) and decreased (blue color) activations during the 2‐back versus 0‐back are presented. For illustrative purposes, maps are thresholded at *p* < .001 FWE corrected. Activations are superimposed on a subject anatomical T1 scan, normalized to the standard MNI space. ACC, anterior cingulate cortex; PCC, posterior cingulate cortex; DLPFC, dorsolateral prefrontal cortex; DMPFC, dorsomedial prefrontal cortex; PC, parietal cortex (superior parietal gyrus and inferior parietal lobule); SMA, supplementary motor area; VMPFC, ventromedial prefrontal cortex. (b) Barplots show the percentage of BOLD signal increase/decrease at peak voxel for 0‐back and 2‐back conditions relative to rest condition. MNI coordinates are indicated. The percentage is calculated over all blocks, that is, safe and threat, for each task difficulty level. Error bars are *SEM*. Light gray = 0‐back, medium gray = 2‐back

Large‐extent clusters of activity were found in the 2‐back versus 0‐back level of the TNT task (Figure 3). We found increased activity in the left and right lateral parts of the middle frontal gyrus belonging to the DLPFC (BA 9), the dorsal ACC (BA 32), the supplementary motor area (SMA, BA 6), and the dorsal premotor cortex (BA 6). A large posterior cluster was also found covering bilateral parietal regions (BA 7/40).

Conversely, some regions were less activated in the 2‐back compared with the 0‐back task. Such effects may represent regional deactivations when participants were engaged in the 2‐back condition. Significant deactivated clusters were observed in the ventromedial prefrontal cortex (VMPFC, with peaks located in BA 10/11) extending to the ventral ACC, the bilateral insula, the bilateral parahippocampal gyrus (PHG), hippocampi and amygdala, and the posterior cingulate cortex. Regional activation and deactivation peaks are reported in Table 1 and illustrated in Figure 3.

**TABLE 1:** Brain activations and deactivations related to task difficulty (*p* < .005, FWE corrected)

Region	Laterality	BA	*X*	*Y*	*Z*	*t*‐value
*2‐back > 0‐back*						
SMA	Left	6	−4	16	48	12.78
Dorsal premotor cortex	Left	6	−36	0	58	10.57
	Right	6	30	−2	54	9.66
DLPFC	Right	9	38	34	30	9.32
	Left	9/46	−38	32	28	9.30
	Left	10	−34	60	10	8.07
	Left	46	−46	20	26	9.74
Dorsal ACC	Right	32	14	22	30	7.64
Anterior insula	Left	13	−28	28	−2	8.73
Angular gyrus	Left	39	−30	−64	36	9.82
Superior parietal gyrus	Left	7	−30	−64	52	8.81
Inferior parietal lobule	Left	40	−50	−44	44	9.42
Precuneus	Left	7	−6	−60	48	9.39
	Right	7	8	−62	52	8.68
Cerebellum	Right		30	−64	−30	10.22
Medial globus pallidus	Left		−14	−4	2	8.67
Thalamus	Left		−8	−24	14	7.77
Dorsal brainstem			2	−36	−16	7.86
*2‐back < 0‐back*						
VMPFC	Medial	11	0	44	−12	9.93
Ventral ACC	Left	10/32	−8	40	−8	9.79
DMPFC	Left	9	−16	54	32	8.63
	Right	8	12	40	56	7.39
Ventral premotor cortex	Left	6	−50	−4	6	7.55
Anterior parietal cortex	Right	40	54	−26	20	9.37
Parahippocampal gyrus	Right	28/35	24	−14	−16	9.52
	Left	35	−24	−22	−18	9.42
Superior temporal gyrus	Right	22	60	0	−2	8.21
Middle temporal gyrus	Left	21	−62	−10	−14	8.19
	Right	21	54	0	−28	7.02
	Left	38	−54	0	−22	6.85
	Left	38	−48	0	−32	6.72
Angular gyrus	Left	39	−50	−66	32	5.94
Amygdala	Right		26	−4	−20	8.22
Posterior insula	Left	13	−40	−10	0	8.47
	Right	13	40	−14	−6	7.47
Dorsal ACC	Medial	24	−4	−6	46	8.73
Dorsal PCC	Medial	23	*−4*	*−50*	28	8.21

Abbreviations: ACC, anterior cingulate cortex; DLPFC, dorsolateral prefrontal cortex; DMPFC, dorsomedial prefrontal cortex; PCC, posterior cingulate cortex; SMA, supplementary motor area; VMPFC, ventromedial prefrontal cortex.

#### 3.4.2 Main effect of auditory stressors

**FIGURE 4:**
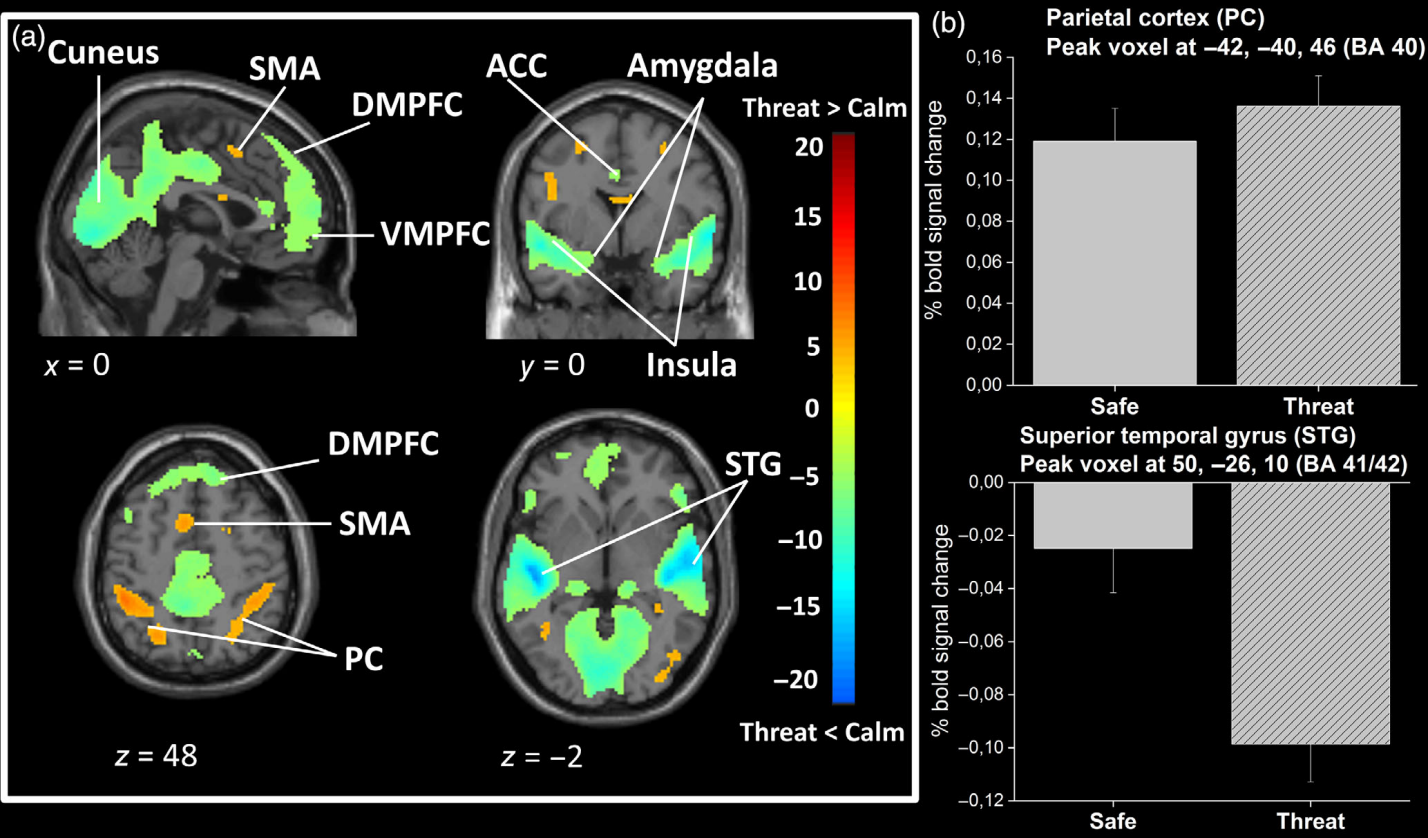
Brain activations and deactivations related to the threat of auditory stressors. (a) Statistical parametric maps illustrating the main effect of threat. The colored bar indicates the *t*‐value (+20 to −20) of the activation height. Cortical areas evidencing increased (orange‐red color) and decreased (green‐blue color) activations during the threat versus safe conditions are shown. For illustrative purposes, maps are thresholded at *p* < .005 FDR corrected. Activations are superimposed on a subject anatomical T1 scan, normalized to the standard MNI space. ACC, anterior cingulate cortex; DMPFC, dorsomedial prefrontal cortex; PC, parietal cortex (inferior parietal lobule); SMA, supplementary motor area; STG, superior temporal gyrus; VMPFC, ventromedial prefrontal cortex (orbitofrontal cortex). (b) Barplots show the percentage of BOLD signal increase/decrease at peak voxel for threat and safe conditions relative to rest condition. MNI coordinates are indicated. The percentage is calculated over all blocks, that is, 0‐back and 2‐back, for each threat level. Error bars are *SEM*. Plain bar = safe, striped bar = threat

When comparing threat versus safe conditions, activations were found in the bilateral parietal cortices (inferior parietal lobule, BA 40) and the SMA (Figure 4). The inverse contrast (threat < safe) revealed large‐extent bilateral deactivated regions induced by the threat condition. The clusters encompassed bilateral superior temporal regions (STG, including primary and secondary auditory cortices) and extended to lower parietal regions and amygdala. Deactivations were also found in the DMPFC (BA 8/9/10), the ventral ACC (32), and the VMPFC (orbitofrontal cortex, BA47). Occipital regions were also deactivated, as well as the posterior cingulate gyrus (BA 31). Activation and deactivation peaks are reported in Table 2 and illustrated in Figure 4.

**TABLE 2:** Brain activations and deactivations related to threat of stressors (*p* < .005, FWE corrected)

Region	Laterality	*Local peak MNI coordinates (mm)*				
		BA	*X*	*Y*	*Z*	*t*‐value
*Threat > safe*						
SMA	Left	6	*−4*	8	50	6.01
Inferior parietal lobule	Left	40	−42	−40	46	7.56
Inferior parietal lobule	Right	40	40	−40	46	5.77
Precuneus	Left	7	−26	*−66*	34	6.06
*Threat < safe*						
DMPFC	Left	9	−10	52	28	5.92
	Left	8	14	44	48	6.05
	Left	10	−18	54	30	7.15
Orbitofrontal cortex	Right	47	50	34	−4	6.85
Superior temporal gyrus	Right	41/42	50	−26	10	21.90
	Left	41	−44	−24	4	19.99
	Left	22	−62	−28	8	15.79
	Right	22	60	−6	6	13.92
Dorsal ACC	Left	24	−4	−12	42	6.97
Anterior insula	Left	13	−38	−20	10	8.81
	Right	13	42	−16	10	8.83
Dorsal PCC	Left	31	−6	−42	36	8.52
Lingual gyrus	Left	18	−4	−84	−4	9.67
Cuneus	Left	18	−6	−96	8	9.02
Amygdala	Left		−28	2	*−24*	7.05

Abbreviations: ACC, anterior cingulate cortex; DMPFC, dorsomedial prefrontal cortex; PCC, posterior cingulate cortex; SMA, supplementary motor area.

#### 3.4.3 Task‐based connectivity related to threat of auditory stressors

**FIGURE 5:**
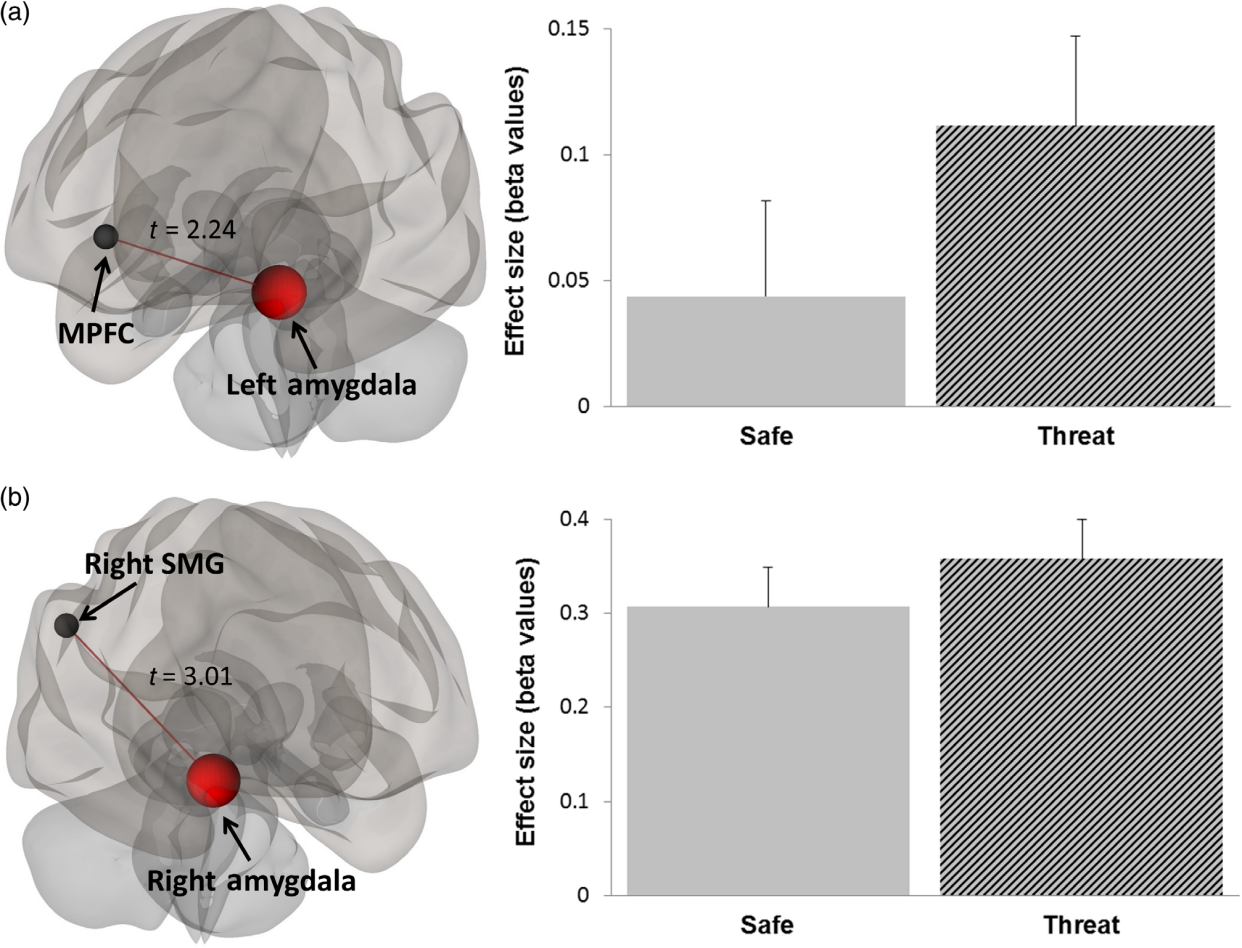
ROI‐to‐ROI connectivity increases when comparing threat of auditory stressors and safe conditions (thresholded at *p* < .05 FDR‐corrected). (a) Higher connectivity between left amygdala and medial PFC ROIs during threat; (b) Higher connectivity between right amygdala and right supramarginal gyrus ROIs during threat. Barplots show average beta estimates of ROI‐to‐ROI connectivity in the group for safe and threat conditions. Error bars indicate *SEM*

The main effect of auditory stressors evidenced significant deactivations in the auditory cortex and in limbic regions belonging to the SN, suggesting inhibition of these regions through top‐down control to reduce the impact of stressors and for emotional regulation. We used bilateral Heschl's gyrus and amygdala atlas ROIs (from the CONN toolbox) as seeds to investigate functional connectivity with all other atlas ROIs. We then compared the connectivity strength between threat and safe conditions (irrespective of the level of task difficulty). At a threshold of *p* < .05 FDR‐corrected, auditory ROIs showed significant connectivity with dorsal prefrontal and parietal regions, although no significant difference in connectivity related to threat could be evidenced. Functional connectivity of amygdala regions with dorsal regions was also significant, and was increased during the threat of stressors (Figure 5, *p* < .05 FDR‐corrected). More precisely, the connectivity between the right amygdala and right inferior parietal cortex (supramarginal gyrus) and the connectivity between the left amygdala and the MPFC were higher during threat (*t* = 3.04, *p* = .007 FDR‐corrected for amygdala‐parietal and *t* = 2.24, *p* < .036 FDR‐corrected for amygdala‐prefrontal connectivity).

#### 3.4.4 Interaction between mental workload and auditory stressors

**FIGURE 6:**
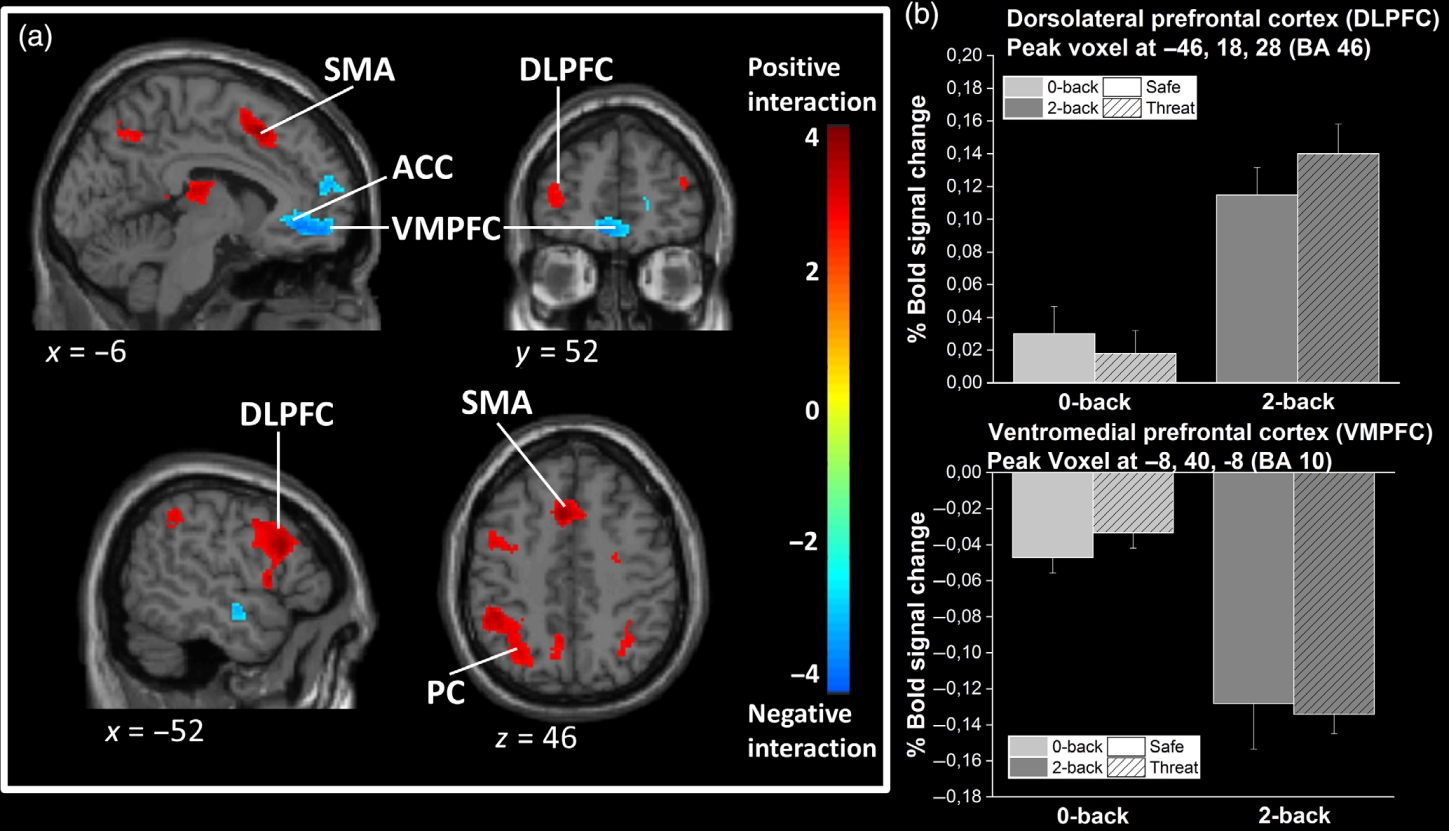
Brain activations and deactivations evidenced in the Difficulty × Threat interaction. (a) Statistical parametric maps illustrating the interaction effects between mental workload and threat of stressors. The colored bar indicates the *t*‐value (+4 to −4) of the activation height. Cortical areas evidencing positive (red color) and negative (blue color) interactions are shown. Maps are thresholded at *p* < .05 FDR‐corrected. Activations are superimposed on a subject anatomical T1 scan, normalized to the standard MNI space. ACC, anterior cingulate cortex; DLPFC, dorsolateral prefrontal cortex; PC, parietal cortex; SMA, supplementary motor area; VMPFC, ventromedial prefrontal cortex. (b) Barplots show the percentage of BOLD signal increase/decrease at peak voxel for each of the four experimental conditions relative to rest condition. MNI coordinates are indicated. Error bars are *SEM*. Light gray = 0‐back, medium gray = 2‐back, plain bar = safe, striped bar = threat

Mental workload‐related increases of activity were more important during the threat compared with the safe condition, that is, positive Difficulty × Threat interaction, in the bilateral superior parietal cortices, in the SMA (Figure 6) and also in two clusters located in the DLPFC (BA 9/46). Conversely, load‐related decreases of activity were more pronounced during the threat condition, that is, negative Difficulty × Threat interaction, in the VMPFC and bilateral hippocampus. Activation and deactivation peaks are reported in Table 3 and illustrated in Figure 6.

**TABLE 3:** Brain activations related to task difficulty × threat of stressors interaction (*p* < .005, FWE corrected)

Region	Laterality	*Local peak MNI coordinates (mm)*				
		BA	*X*	*Y*	*Z*	*t*‐value
*Positive interaction*						
SMA	Left	6	−6	18	48	4.84
	Right	6	8	18	46	3.71
	Left	6	−8	10	56	3.84
DLPFC	Left	9	−56	16	30	4.49
	Left	46	−46	18	28	4.78
Inferior frontal gyrus	Left	44	−52	12	8	3.89
Inferior parietal lobule	Left	40	−50	−44	44	4.28
Superior parietal lobule	Left	7	−28	−68	44	3.98
*Negative interaction*						
VMPFC/ventral ACC	Left	10/32	−8	40	−8	4.69
Hippocampus	Left		*−24*	*−28*	−14	4.12
	Right		26	*−12*	*−14*	4.34

SMA, supplementary motor area; DLPFC, dorsolateral prefrontal cortex; VMPFC, ventromedial prefrontal cortex; ACC, anterior cingulate cortex.

#### 3.4.5 Brain activity co‐varying with heart rate and pupil diameter based on parametric modulation analyses

The increase of brain activity due to mental workload (2‐back > 0‐back) did not significantly co‐vary with heart rate or pupil diameter (threshold of *p* < .005). During expectation of auditory stressors (threat > safe), the increase of activity in several brain regions was positively correlated with heart rate and pupil diameter. Heart rate variations were notably associated with modulations of activity in the occipital lobe, hippocampus, precuneus or ventral posterior cingulate cortex (Table 4). Pupil diameter variations under the threat condition were associated with higher dorsal prefrontal regions (BA 9/8/10) and occipital activity (Table 4). It is worth noting that pupillary changes track cognitive workload at a fine‐grained level. Therefore, as mental workload induced variations in tonic pupil diameter between blocks, it would also be interesting to perform a point‐by‐point correlation analysis (i.e., at the fMRI temporal resolution) between the phasic pupillary response and brain activity. However, due to technical difficulties, the pupillary and BOLD signals were point‐by‐point synchronized only in 12 participants. A further investigation of this sub‐sample might reveal correlations between workload‐induced changes in tonic pupil diameter, phasic pupillary response, and brain activations.

**TABLE 4:** Brain activations positively correlated with heart rate and pupil diameter during threat of stressors (threat > safe, *p* < .005, uncorrected)

Region	Laterality	*Local peak MNI coordinates (mm)*				
		BA	*X*	*Y*	*Z*	*t*‐value
*Heart rate and threat of stressors*						
Middle occipital gyrus	Left	19	*−40*	*−84*	12	*3.80*
Hippocampus	Right		*30*	*−26*	*−6*	3.60
Precuneus	Right	7	24	*−66*	*36*	3.50
Ventral PCC	Right	30	8	*−52*	10	3.37
Superior temporal gyrus	Right	40	*56*	*−66*	28	3.30
Middle occipital gyrus	Left	19	−44	−74	20	3.06
*Pupil diameter and threat of stressors*						
Fusiform gyrus	Left	37	−32	−58	−8	3.51
Anterior PFC^a^	Right	10	24	60	18	3.50
DMPFC	Right	8	12	30	58	3.40
DLPFC	Right	9	46	22	34	2.97
Inferior occipital gyrus	Left	18	−32	−90	−12	2.95

Abbreviations: DLPFC, dorsolateral prefrontal cortex; DMPFC, dorsomedial prefrontal cortex; PCC, posterior cingulate cortex.

^a^ The *p*‐value survived small volume correction (SVC) within a sphere of 5‐mm radius in the right DLPFC centered at MNI coordinates: [24, 45, 18] (DiNuzzo et al., 2019).

## 4 DISCUSSION

We investigated the way the brain deals with an important mental workload and/or a threat situation. We speculated that our TNT n‐back task with additional cognitive workload (mental calculation) would deteriorate under high workload and we expected greater involvement of the ECN regions. Overall, the slight decrease in performance indicated that task difficulty actually increased mental workload and provoked an enhanced activity in the ECN, in particular the lateral prefrontal and parietal regions, along with a disengagement of the DMN, including the medial prefrontal cortex. Task difficulty also clearly affected subjective difficulty and autonomic activity with increased heart rate and pupillary diameter.

We expected a moderate decrease of performance with the stressors, and a large decrease of performance related to the ECN disengagement when high task difficulty was combined with the stressors. This scenario turned out to be wrong despite increased subjective anxiety with threat. Preservation of performance under threat might be due to efficient cognitive strategies used by participants, as indexed by important changes in brain activity that accompanied the threat condition: an increase of the activity in the ECN regions (lateral prefrontal and parietal cortices) along with deactivation in SN regions (i.e., amygdala, hippocampus), as in the study by Clarke and Johnstone (2013). We also found large deactivations of the superior temporal regions, suggesting attentional filtering mechanism of the aversive stimuli. When high workload was combined with the stressors, we found a pattern quite similar to those observed with the high workload, with extra activation in the ECN regions along with deactivation of the VMPFC.

### 4.1 Increased mental workload was associated with reduced performance, higher ECN activity, and increased autonomic response

Our behavioral results indicated that increased mental workload was associated with a decrease in n‐back task accuracy and an increase in reaction times. Our extremely difficult task elicited activity in the fronto‐parietal network, including the DLPFC, the dorsal ACC, and the parietal cortex. These regions are part of the ECN (Shenhav et al., 2017), which is considered to be more activated when an individual must perform a task demanding effort, sustained attention or maintenance of information in WM, and is less activated when performing more habitual behaviors (Shenhav et al., 2017). ECN involvement has been notably reported during n‐back tasks (Jansma, Ramsey, Coppola, & Kahn, 2000; Luo et al., 2014; Schmidt et al., 2009). In our study, a large recruitment of the SMA and the premotor cortex (BA 6) was also found with increased load in WM. SMA is considered to be a modality independent circuitry sustaining WM processes (Schumacher et al., 1996). This region was also markedly activated during n‐back performance in the Schmidt et al. (2009) study. Moreover, the SMA and premotor cortex have been associated with mental calculation (Hanakawa et al., 2002). In our 2‐back TNT condition, participants had to compute an exact arithmetic result to compare it with the target obtained two trials before. In the 0‐back condition that implied to compare the arithmetic result with “50,” it is likely that estimating the order of magnitude of the result was sufficient to complete the task. Accordingly, higher workload in our task resulted in increased activation of motor and ECN regions (Gruber et al., 2001), that is, an extended fronto‐parietal network.

Besides ECN involvement, several cortical areas owning to the DMN (Fransson & Marrelec, 2008; Greicius et al., 2003; Raichle et al., 2001; Ward et al., 2014) showed large deactivations in response to increased mental effort, including the medial PFC (i.e., DMPFC and VMPFC), the PCC, the ventral ACC, and the middle temporal gyrus. The DMN, active during awake rest, would be implicated in the generation of spontaneous task‐unrelated thoughts (Mason et al., 2007) whose occurrence can represent a source of internal distraction (Hinds et al., 2013). It has been shown that regions involved in the DMN could also evidence increased activity when the task is too difficult, reflecting a disengagement from the task (Buckner, Andrews‐Hanna, & Schacter, 2008). Greater involvement of the DMN can thus negatively affect the performance of a demanding task (Smallwood, Beach, Schooler, & Handy, 2008), including driving a vehicle (Galéra et al., 2012; He, Becic, Lee, & McCarley, 2011) or flying a plane (Casner & Schooler, 2014; Durantin, Dehais, & Delorme, 2015). In our study, regions owning to the SN were also deactivated with task difficulty, in particular the amygdala and the insula. Taken together, our data, therefore, suggest efficient allocation of neural resources to the ECN at the expense of the DMN and the SN, that is, efficient brain mechanisms to perform the complex 2‐back task.

Regarding autonomic measures, heart rate and pupil diameter were increased during the 2‐back condition, indicating physiological arousal with a shift of the balance of the autonomic nervous system toward a sympathetic dominance (Simpson et al., 2001). High workload activated the dorsal brainstem, which may reflect additional locus coeruleus (LC) and ventral tegmental area (VTA) nuclei involvement. LC and VTA phasic response may have induced transient increases in catecholamine levels, thereby enhancing DLPFC activity and facilitating executive and WM processes to maintain performance to respond to increased task difficulty (Arnsten, 2009; Sara, 2009; Yuen et al., 2009). In addition, an increase of catecholamine brain levels induces stimulation of the sympathetic branch of the autonomic nervous system (Sara & Bouret, 2012). Our observation of higher heart rate and pupil size in the 2‐back trials is therefore consistent with phasic catecholamine release.

### 4.2 Task performance was preserved despite the stressors: SN disengagement and inhibition of the auditory processing

The threat condition resulted in significantly higher anxiety ratings, although it did not elicit significant behavioral or autonomic modifications. Preservation of performance under stressors in our complex WM task is coherent with several previous studies that have used single‐letter‐ or digit‐n‐back tasks (Clarke & Johnstone, 2013; Cousijn et al., 2012; Duncko et al., 2009; Porcelli et al., 2008; Qin et al., 2009) as well as similar highly demanding WM task (Mandrick et al., 2016). Our neuroimaging results suggest that maintaining performance under stress induction was likely conditioned by additional allocation of neural resources within the ECN toward the cognitive task (Eysenck & Calvo, 1992; Mandrick et al., 2016), inhibition of the STG to reduce auditory stressor processing, and inhibition of limbic regions of the SN for emotional regulation.

The threat condition was dominated by significant deactivations in the STG and cuneus, extending to the posterior cingulate gyrus. Deactivations were also observed in the dorsal and ventral medial PFC, the ACC, the insula and the amygdala. Deactivation of the STG most certainly reflects inhibition of auditory processing during the expectation of aversive loud sounds. Deactivation of brain regions has been probably under‐considered in the past because of the inclination to deal primarily with the neural activation effects induced by stimulation, rather than with any possible decrements in neural activity. Yet, it is well known that humans have the capacity to selectively enhance or inhibit sensory brain regions via top‐down attentional mechanisms (Mozolic et al., 2008). Our result may thus be interpreted in several ways. First, the focal task (i.e., the TNT) may have consumed most attentional resources, provoking a reduced distractor effect (Hu, Bauer, Padmala, & Pessoa, 2012) since attention was shifted away from the aversive auditory stimuli (Pessoa, McKenna, Gutierrez, & Ungerleider, 2002). Consequently, less attention was paid to “expect” and to process the sounds. An additional and more cognitively active explanation is that inhibition of auditory processing may have contributed to filter the unwanted noises. Interestingly, top‐down inhibitory mechanisms have been proposed to explain the attenuation of unpleasant sounds in the context of tinnitus habituation (Rauschecker, Leaver, & Mühlau, 2010). Such selective sensory inhibition is thought to take place over long periods of time and following repetitive aversive stimulation. Although this long‐term neural adaptation could not occur in our experiment, it is possible that similar initial control mechanisms helped to protect from its stressful effects. Note, however, that the useful deactivations of large temporal regions are to be interpreted within a specific context, since “ignoring” a stressful stimulus is not always a good strategy. When extreme, this phenomenon has been described as inattentional deafness and is subtended by inhibitory brain mechanisms of the auditory processing (Dehais, Roy, & Scannella, 2019; Giraudet, St‐Louis, Scannella, & Causse, 2015). While it may be useful when the aversive auditory stimulus is an unwanted disturbance, it becomes dangerous when it is a security alarm that is ignored. This points to the complexity of using physiological measures to assist the operator, and the need to go deeper into the understanding of these mechanisms.

Furthermore, several regions of the SN (and in particular the VMPFC, anterior insula, and amygdala) were deactivated during the threat condition, as observed in other studies (Clarke & Johnstone, 2013; Pruessner et al., 2008; Simpson et al., 2001), suggesting implication of emotional regulation mechanisms during threat. Consistent with the latter interpretation, the functional connectivity between dorsal prefrontal/parietal regions and amygdala was increased during the threat condition. In rodents, the MPFC and amygdala have a major role in stress‐related behaviors, and MPFC has an important role in the regulation of amygdala response to emotional stimuli (Andolina, Maran, Valzania, Conversi, & Puglisi‐Allegra, 2013). Such regulation mechanisms through top‐down control exerted by the PFC on the amygdala have been reported in humans, during concurrent cognitive and emotional tasks (Loos et al., 2020; for review see Okon‐Singer, Hendler, Pessoa, & Shackman, 2015). Consistently, our functional connectivity results suggest that, in our experiment, top‐down control on SN regions may have occurred during threat of stressors. In particular, the deactivation of the anterior insula may explain the absence of visible effects of the threat condition on heart rate and pupil size measures, despite self‐report of increased anxiety.

When stressors were concomitant with high workload, performance remained preserved. We observed that deactivation of the VMPFC was greater (i.e., negative interaction effect). Therefore, deactivation of this region may be critical for optimal cognitive performance when facing both high load context and the threat of stressors. Bilateral hippocampus deactivations can be also linked to decreased activity in the SN. Previous studies found that the SN plays a critical role in mediating the interaction between emotion perception and executive control (Luo et al., 2014). Moreover, we observed supplementary activity in the SMA, in the DLPFC, as well as in the left parietal cortex (i.e., positive interaction effect). These regions were also implicated in the TNT performance in the safe condition. This increased brain activity in task related regions suggests that additional cognitive resources were engaged to preserve performance. Similar increases of activity in the ECN regions under stressors were found in previous studies (Clarke & Johnstone, 2013; Porcelli et al., 2008) and arithmetic tasks (Dedovic et al., 2009).

Interestingly, we found that activity in dorsal medial and lateral PFC regions was related with pupil dilation under stress induction. Pupil dilation dynamics has been closely associated with LC activity (both tonic and phasic, Joshi, Li, Kalwani, & Gold, 2016) and may be a reliable proxy measure of norepinephrine (NE) release and sympathetic involvement. Activity in the dorsal frontal gyrus was found to be related with pupil size during rest (DiNuzzo et al., 2019), thus reflecting variations of tonic NE brain levels. Therefore, in our study, pupil dilation likely indexed the increased activity in the dorsal PFC regions implicated in the cognitive task and enhanced under the threat of the stressors, as a result of a tonic increase in brain catecholamines.

Altogether, our results showed that facing high load context and the threat of stressors could be characterized by a specific pattern of PFC activity. Increased activation of the ECN would play a role for the maintenance of cognitive performance, while diminishing sensory processing. This would be explained by exhaustion of attentional resources or inhibition, that helped modulating the impact of the auditory stressors. Without being contradictory to these mechanisms, successful preservation of performance may have been possible in our experiment since actual stress level was probably relatively moderate, as inferred from anxiety ratings (i.e., 5.10 in the “2‐back threat” condition, on a 0–10 scale) and the lack of increased sympathetic activity, in particular the heart rate that is known to be sensitive to acute stress (Yao et al., 2016). It has been shown that passive exposure to loud aversive sounds induces significant activations in the SN and dorsal brainstem, thus clearly evidencing a marked stress response (Zald & Pardo, 2002). However, the threat of intermittent aversive sounds, as used in our study, may have been less stressful than direct exposure to permanent sounds, but we aimed at eliminating possible distraction effects. One must acknowledge that despite the subjective and neuroimaging results that suggest efficient cognitive strategies, the lack of behavioral and physiological effects of the stressor can raise a doubt on the effectiveness of the stress manipulation. Indeed, an intense level of stress would have probably provoked an increased level of emotional arousal indexed by a heightened autonomic nervous system activity. We can also postulate that a more efficient and intense stressor would have degraded task performance and elicited different patterns of brain activations, in particular with the recruitment of regions pertaining to the SN.

### 4.3 How far do we need to go to observe cognitive disengagement?

Compared with previous studies on concomitant cognitive task and acute stress, our primary goal was to increase the mental effort with a task that combined WM and mental calculation, two cognitive processes that rely on the ECN. We did not observe a drop of performance or a decrease in ECN function when combined with stress. There are three ways to induce cognitive disengagement in such experimental design: increasing the load, increasing the stress or increasing both of them. The increase of the cognitive load is limited: the task would have to be hardly feasible without stress and become unfeasible when stress is administered. We think that this ceiling of difficulty might have been reached with the very difficult TNT. However, participants did adapt with the threat of aversive sounds, and performance at the task was similar with and without stress. Increasing the level or changing the nature of stress, may therefore, in this context, be the solution. Given the mentioned low efficiency (and possibly zero efficiency in some participants) of the threat of aversive sounds, for future experiments, we would suggest turning to other types of stressors. For example, psychosocial stress is difficult to filter out, contrarily to auditory stressors, and have been proven to reduce performance (Jiang & Rau, 2017; Schoofs et al., 2008). These scenarios are not easy to implement in an fMRI experiment because to be efficient, they must be very realistic. This would be however a great challenge and an interesting path that may well fit with the high levels of stress that airplane pilots experience due to high hierarchical pressure, huge financial outcomes, and even life and death responsibility.

It is also important to note that experimental 2 × 2 designs combining factors of workload and stress, such as used in our and others' studies (Clarke & Johnstone, 2013; Cousijn et al., 2012; Duncko et al., 2009; Jiang & Rau, 2017; Porcelli et al., 2008; Qin et al., 2009; Schoofs et al., 2008), remain approximate to selectively point out effects of stress on cognition. Indeed, cognitive task difficulty itself can induce acute stress. In our study, both levels of subjective difficulty and anxiety were increased by higher task load, and one might argue that task‐related stress might have participated in the observed shift toward sympathetic dominance. Indeed, increased workload in a challenging task can elicit a mixture of mental workload and acute stress since the task can be emotionally challenging (Parent et al., 2019). Raised anxiety related to task performance was evidenced by Simpson et al. (2001). The authors assumed that anxiety is a likely accompaniment of most demanding cognitive tasks conducted in a laboratory or imaging settings, especially during naïve task performance. As a consequence, areas of the brain concerned with emotion may change in concert with those more directly concerned with the cognitive aspects of the tasks. However, in our study, brain regions related to the 2‐back condition largely suggest that task‐related stress remained moderate as no clear activation of the SN was evidenced. Conversely, the opposite pattern was observed, that is, deactivation of the SN.

### 4.4 Perspectives for neuroergonomics

Complex and safety‐critical activities such as piloting can lead to an increase of both mental workload and acute stress, whose effects can engender poor cognitive performance, bad decisions (Maier, Makwana, & Hare, 2015), and eventually accidents. In this context, the maintenance of optimal cognitive performance is a constant challenge. Here we observed that high load context and the threat of stressors may be overcome thanks to compensatory mechanisms. The balance of high activity in the ECN combined with reduced activity in the SN and the DMN were markers of efficient brain mechanisms and preserved performance. Conversely, with more load and more intense stress, the disruption of this balance may be used as a marker of cognitive disengagement and disability to appropriately face an emergency. As in situ measurements of mental workload are expanding rapidly with the development of field‐deployable neuroimaging tools like functional Near‐Infrared Spectroscopy (fNIRS), caution must be taken, especially when safety‐related decisions are supported by physiological objective measurements. Our results confirmed that increased activity in the DLPFC, belonging to the dorsal fronto‐parietal network, successfully indexed effortful (but efficient) mental activity. This effortful mental activity was accompanied by a reduction of the DMN activations, including the DMPFC. During combined high workload and threat of stressors, greater deactivations were observed in the VMPFC. Using frontal fNIRS, the DLPFC/medial PFC cerebral blood flow ratio could thus efficiently index mental workload and emotional regulation.

Regarding autonomic measures, increased heart rate and pupil diameter in the more demanding task confirmed that variations in heart rate and pupil diameter can be reliable indexes of mental workload during task performance. This is consistent with a large body of literature (Kahneman & Beatty, 1966; Shine et al., 2016; van der Wel & van Steenbergen, 2018), including studies addressing critical domains like piloting (Peysakhovich et al., 2015; Roscoe, 1992; Wilson, 2002). Among promising applications, we can cite the evaluation of systems during the certification process via the objective evaluation of the mental workload and stress they can generate, adaptive automation (Byrne & Parasuraman, 1996), crew monitoring in the cockpit, or driver monitoring in autonomous vehicles.

## 5 CONCLUSION

Our results have highlighted the cerebral and physiological signature of high mental workload and threat of auditory stressors. Mental workload was characterized by a marked activation of the ECN along with disengagement of the DMN and SN. Cardiovascular activity and pupillary diameter were good indicators of mental workload, with possible links between pupil diameter variations and superior frontal activity. Threat of auditory stressors did not result in changes in autonomic activity or task performance but increased recruitment of task‐related regions (in particular the ECN, with lateral prefrontal and parietal regions, as well as the SMA), decreased activation of the medial PFC, and deactivation of auditory and SN regions were observed, suggesting adaptive brain mechanisms to focus on the task and inhibit processing of the stressors and regulate emotion. During concomitant high workload and stressors, we observed a similar pattern with higher recruitment of task‐related regions and decreased activation of the medial PFC.

Our results critically underline that extra recruitment of task‐related regions, in particular in the lateral prefrontal and parietal regions, and a pattern of PFC activations/deactivations, that is, increased lateral PFC and decreased medial PFC activity, may represent a relevant marker of preserved performance and successful management of both high load and threat of stressors. We can however assume that stress levels were moderate in our experiment and more intense stress may have finally resulted in different patterns of activations and a performance decrement.

## CONFLICT OF INTEREST

The authors declare no conflicts of interest.

## Data Availability

fMRI data are available at the NeuroVault data repository: neurovault.org. URL: https://identifiers.org/neurovault.collection:9469. Behavioral data are available from the authors upon request.
